# Enhancement of a-IGZO TFT Device Performance Using a Clean Interface Process via Etch-Stopper Nano-layers

**DOI:** 10.1186/s11671-018-2571-9

**Published:** 2018-05-29

**Authors:** Jae-Moon Chung, Xiaokun Zhang, Fei Shang, Ji-Hoon Kim, Xiao-Lin Wang, Shuai Liu, Baoguo Yang, Yong Xiang

**Affiliations:** 1 School of Materials and Energy, University of Electronic Science and Technology of China, 2006 Xiyuan Ave, West High-Tech Zone, Chengdu, 611731 Sichuan China; 2 Chongqing BOE Optoelectronics Technology CO., LTD, Chongqing, 400718 China; 3 The 41st Research Institute of CETC, Qingdao, 266555 Shandong China

**Keywords:** Displays, a-IGZO, Thin film transistors, Etch-stopper, Reproducibility, Reliability

## Abstract

To overcome the technological and economic obstacles of amorphous indium-gallium-zinc-oxide (a-IGZO)-based display backplane for industrial production, a clean etch-stopper (CL-ES) process is developed to fabricate a-IGZO-based thin film transistor (TFT) with improved uniformity and reproducibility on 8.5th generation glass substrates (2200 mm × 2500 mm). Compared with a-IGZO-based TFT with back-channel-etched (BCE) structure, a newly formed ES nano-layer (~ 100 nm) and a simultaneous etching of a-IGZO nano-layer (30 nm) and source-drain electrode layer are firstly introduced to a-IGZO-based TFT device with CL-ES structure to improve the uniformity and stability of device for large-area display. The saturation electron mobility of 8.05 cm^2^/V s and the *V*_th_ uniformity of 0.72 V are realized on the a-IGZO-based TFT device with CL-ES structure. In the negative bias temperature illumination stress and positive bias thermal stress reliability testing under a ± 30 V bias for 3600 s, the measured *V*_th_ shift of CL-ES-structured device significantly decreased to − 0.51 and + 1.94 V, which are much lower than that of BCE-structured device (− 3.88 V, + 5.58 V). The electrical performance of the a-IGZO-based TFT device with CL-ES structure implies that the economic transfer from a silicon-based TFT process to the metal oxide semiconductor-based process for LCD fabrication is highly feasible.

## Background

Thin film transistor (TFT) backplane with higher resolution and larger panel size is highly desired in the flat plane display industry. Semiconductor material with a high electron mobility is crucial to improve the performance of TFT backplane. In particular, a metal oxide semiconductor-based TFT backplane is considered as a promising candidate to overcome the limitation of silicon-based TFT backplane in terms of mechanical flexibility and electron mobility [1–4]. Although a metal oxide semiconductor-based TFT backplane shows promising properties, the process method with a low-cost process for both large-scale deposition for industrial application is still needed [5].

Amorphous indium-gallium-zinc-oxide (a-IGZO) is an excellent metal oxide semiconductor with a high saturation electron mobility (~ 5–10 cm^2^/V s) and a low off-current (< 10 pA) [6–10]. The common industrial production method for a-Si:H-based TFT backplane is five-mask-back-channel-etched (BCE) process. However, a-IGZO nano-film has a very low chemical resistance to the typical etchants currently used in the BCE process. Especially, a-IGZO nano-films would be completely etched in few seconds when they are exposed to Al etchant composed of phosphoric acid, nitric acid, and acetic acid [11–13]. This uncontrollably fast etching hinders the adoption of BCE process for a-IGZO-based TFT backplane. To utilize a-IGZO in BCE-structured backplane, Cu wiring technology has been developed, as the etchant used in Cu wiring process, which is based on H_2_O_2_, is much milder to a-IGZO nano-film than the ones used in Al wiring [11, 13]. Unfortunately, a-IGZO nano-film is still damaged during Cu wiring process even when milder etchant is used. Even milder etchants cause damage on the surface of a-IGZO nano-film that forms back channel of TFT devices. These damages cause collapse in the stoichiometric molecular composition ratio near the surface of a-IGZO nano-film, leading to the aggravation of uniformity in large-area display and TFT device reliability. To date, conventional six-mask-etch-stopper (CV-ES) process is developed to fabricate a-IGZO-based TFT backplane with etch-stopper-layer (ESL) structure [14, 15]. However, this six-mask ES process may lead to a negative economic feasibility. Moreover, this increased number of thin film layers would increase the inter-layer overlap area and result in the increased parasitic capacitance and decreased opening ratio [16–18]. Although five-mask ES process that produces TFT backplane using half-tone and lift-off technology has been reported recently, this process is not accessible for the production of a-IGZO-based TFT backplane, as their active layer surface is still exposed to process chemicals such as stripper and photoresist in the last step, which may cause considerable contamination to a-IGZO, thus reducing the device quality and the production yield [19–21]. Therefore, the industrial production method for a-IGZO-based TFT backplane with highly uniformity and stability remains challenging.

In this paper, we propose a clean five-mask ES process (CL-ES) via introducing ESL for fabrication of a-IGZO-based TFT backplane. This newly developed CL-ES process is highly compatible with the existing process for BCE device. This CL-ES process is designed to have the equal masks to that of BCE process, which ensures a negligible loss of productivity of existing AM-LCD TFT backplane FAB. a-IGZO-based backplane produced using CL-ES process deposits gate insulator, IGZO nano-layer, and ES nano-layer sequentially, then forms a new ESL mask through dry-etch method. This could prevent the contamination of a-IGZO nano-layer and their interface from etchant, stripper, and solvent. This newly formed nano-mask helps improve the uniformity and stability of TFT device. Compared to conventional BCE-structured device, a-IGZO-based device with CL-ES structure shows enhanced electrical performances, namely a higher saturation electron mobility, a high opening ratio, and a low power consumption.

## Methods/Experimental

### Fabrication of a-IGZO-based TFT Backplane

**Fig. 1:**
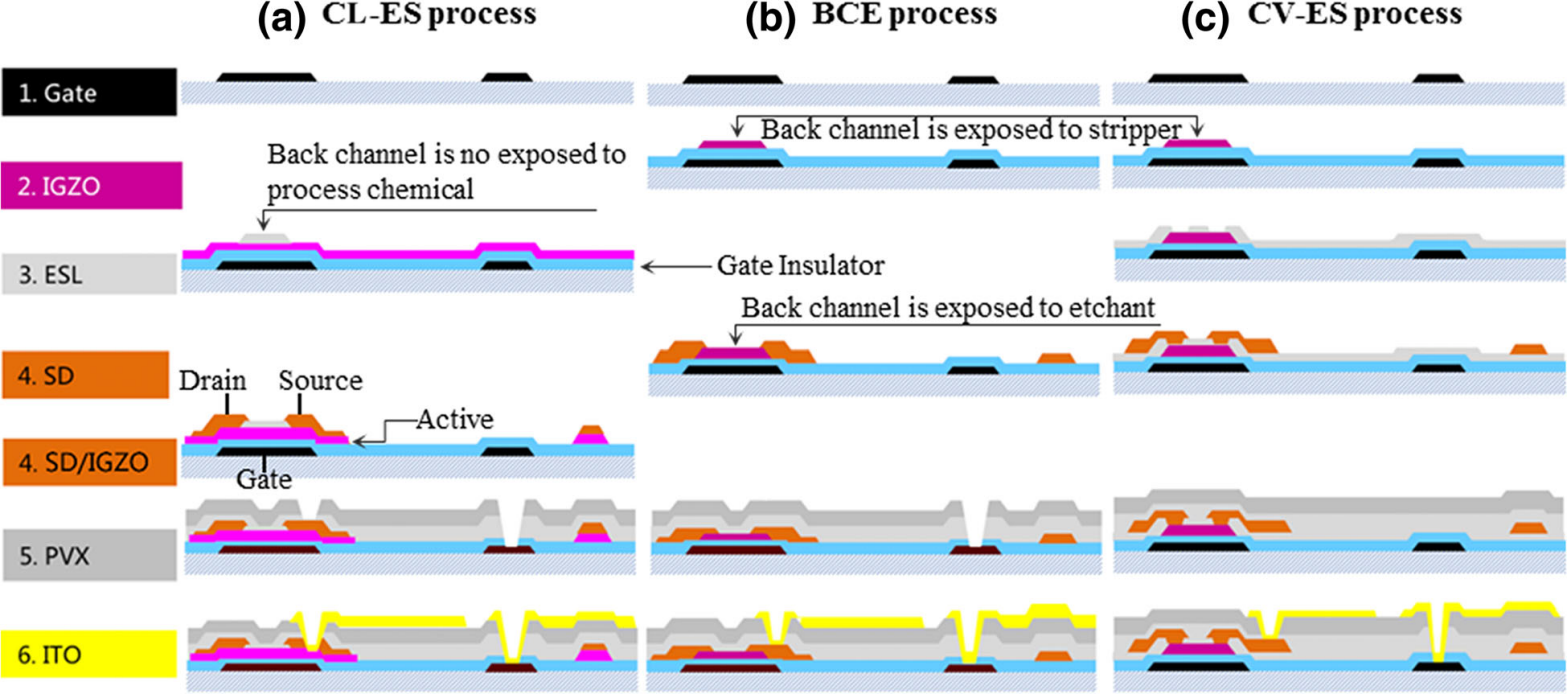
(Color online) Schematics of **a** CL-ES, **b** BCE, and **c** CV-ES processes

The a-IGZO-based TFT backplane with ES structure fabricated via CL-ES process was as follows (Fig. 1).

Firstly, double layer (Mo/Cu:30 nm/250 nm) was used for gate electrode as it has a reasonably low resistivity. Then, gate insulator, silicon nitride (SiNx)/silicon oxide (SiOx) (300 nm/100 nm), was deposited by plasma-enhanced chemical vapor deposition (PECVD) method. This SiNx film is designed to prevent oxidation of Cu metallizing and diffusion of Cu ion into the gate insulator. Subsequently, a SiOx thin film was deposited. The deposition conditions of PECVD SiOx film were 17-KW RF power, 1000 mTorr pressure, 1:55 SiH_4_/N_2_O gas ratio, and 350 °C temperature. Then, a-IGZO nano-film was deposited to 30 nm using dc rotary magnetron sputter. The target had the diameter of 171 mm while the composition was In_2_O_3_:Ga_2_O_3_:ZnO = 1:1:1 mol%. The sputtering parameters for the a-IGZO nano-film were system base pressure of 5~ 9 × 10^−7^ Torr, rf sputtering power of 10 KW, sputtering pressure of 5-mTorr Ar/O_2_ gas mixture (85% Ar-15% O_2_). The deposition temperature is at room condition. Produced a-IGZO film is annealed at 330 °C for 1 h in clean dry air environment.

Secondly, etch-stopper (ES) nano-layer (SiOx) was deposited using PECVD method. ES nano-layer is simultaneously deposited to prevent contamination in a-IGZO layer. As in BCE process, there is no protective layer for a-IGZO nano-film before S/D electrode patterning process, surface contamination, and damage on a-IGZO nano-film by S/D etchant when forming TFT channel is unavoidable. ES nano-layer in CL-ES process can effectively protect TFT channel from external contamination and damage. The ES nano-layer was deposited to a thickness of 100 nm. The deposition conditions of SiOx thin film were 17-KW RF power, 1000 mTorr pressure, 1:66 SiH_4_/N_2_O gas ratio, and 240 °C temperature. The ES nano-mask produced was etched by dry etching and patterning. During the etching process, CF_4_ and O_2_ gas were supplied at a rate of 2000 sccm/800 sccm.

Thirdly, Mo/Cu/Mo was also used for S/D electrode. To select S/D electrode of a-IGZO TFT, the work function difference between metal and a-IGZO was considered to form an Ohmic contact and the low-resistivity materials. As described in the etch-stopper process, during the patterning of ES nano-mask, the a-IGZO nano-films, which are not protected by the etch-stopper layer, are already conducted by being bombarded with CF_4_ plasma. Therefore, Ohmic contact is formed naturally with Mo/Cu/Mo [22]. The S/D layers were deposited in the thickness of 30 nm/300 nm/30 nm with the same sputtering conditions as the gate electrode. In addition, multi-thin film layers of Mo/Cu/Mo and a-IGZO were batch etched using “H_2_O_2_ based Cu etchant containing a fluoride additive” to complete the S/D electrode. The 30 nm of Mo added on top of Cu was formed to prevent oxidation of Cu surface by passivation film (SiOx) in the next process and to prevent plasma damage of Cu surface, during dry etching for passivation hole formation.

Fourthly, passivation film, divided into two kinds of thin film, was deposited using PECVD method. The first passivation was made of SiOx thin film. The thin film was 250 nm thick. The second passivation constituted of SiNx thin film. The thickness of the thin film was 200 nm.

Fifthly, as the pixel electrode, indium tin oxide (ITO) film, which is most commonly used in the display industry, was utilized. The ITO film was 40 nm thick, and dc sputtering was used for the deposition. Then, the final annealing was carried out in a clean dry air environment at 230 °C for 1 h using a hot air oven. The electrical characteristics of manufactured a-IGZO TFTs were measured using Keysight 4082A Parametric Test System. This process will obtain the same number of masks (TN product standard: five masks) as the BCE process, which is widely used in mass manufacturing.

For comparison, a-IGZO-based TFT backplane with BCE structure was fabricated via BCE process.

### Characterization

TFT’s I-V measurement was conducted at room temperature using a semiconductor characteristic analyzer. The analyzing condition to evaluate the TFT’s stability under negative gate bias temperature illumination stress (NBTIS) was as follows. *V*_gs_ and *V*_ds_ were respectively fixed at − 30 and 15 V, and the temperature of the substrate was kept at 60 °C. The luminance for NBITS was set at 5000 cd/m^2^. The duration of the stress for evaluation continued for 3600 s [23]. Positive gate bias thermal stress (PBTS) were tested at a *V*_gs_ of 30 V and a *V*_ds_ of 15 V, and the substrate temperature was set at 60 °C. The duration of the stress for evaluation continued for 3600 s [24].

## Results and Discussion

**Fig. 2:**
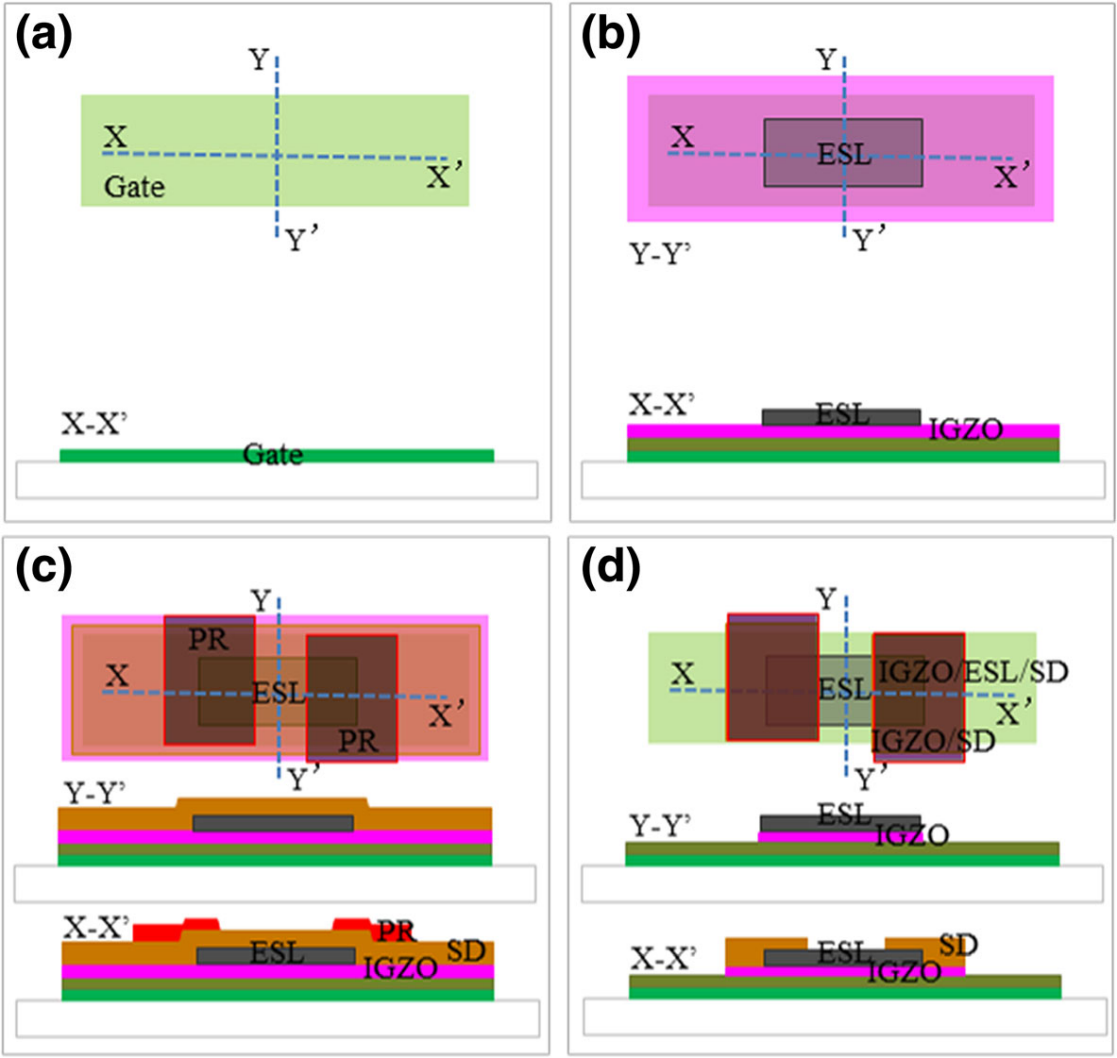
(Color online) Schematics of simultaneous formation method for TFT channel and S/D electrode in CL-ES process. **a** The first step that forms gate electrode. **b** The second step that forms etch-stopper layer. **c** The third step that forms S/D photo pattern. **d** The fourth step that forms S/D electrode and active pattern

**Fig. 3:**
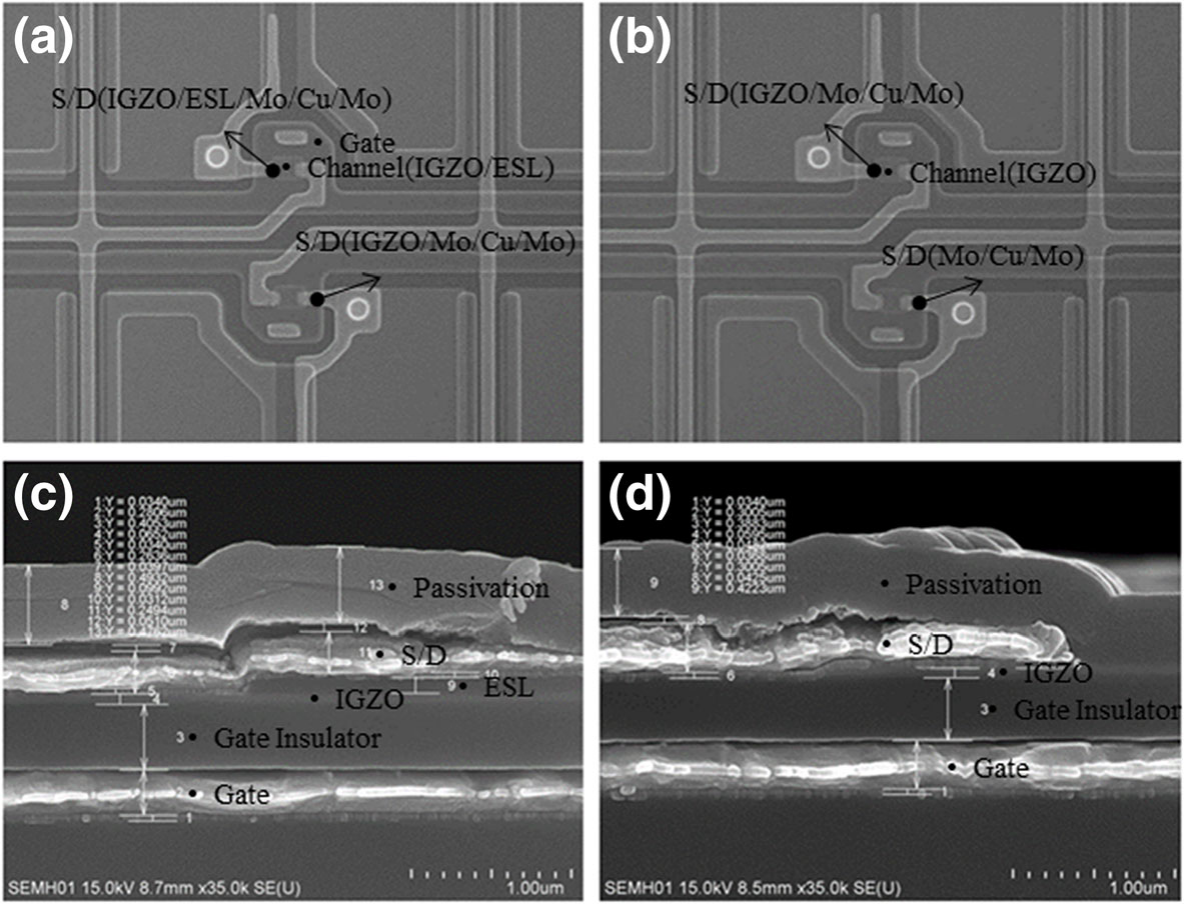
(Color online) SEM images of a-IGZO TFT (**a**, **b** top view; **c**, **d** side view) with CL-ES structure (**a**, **c**) and BCE structure (**b**, **d**)

a-IGZO-based TFT fabricated via CL-ES process shows the same mask number to that of BCE process (Fig. 1). Compared with a-IGZO-based TFT with BCE structure, a-IGZO-based TFT with CL-ES structure shows two advantages: (1) a-IGZO-based backplane produced using CL-ES process deposits gate insulator, a-IGZO nano-layer, and ES nano-layer sequentially, then forms a ESL nano-mask through dry-etch method. This newly formed ESL nano-mask with 100 nm can prevent the exposure of a-IGZO nano-film to etchant, stripper, or photoresist. Therefore, the contamination at inter-layer interfaces is effectively prevented [25]. (2) At the same time, a-IGZO nano-film is not protected by ES layer but bombarded by CF_4_ plasma during the ESL nano-mask formation, thus becomes a conductor. This naturally forms the Ohmic contact between S/D electrode of following process and a-IGZO semiconductor. For another part, a simultaneous etching of S/D and a-IGZO nano-layer can be one overlay allowance of ESL-(a-IGZO+S/D metallization) layer, which could decrease the two overlay process error of the a-IGZO-ESL and ES-S/D metallization layer in the conventional ESL process (Fig. 2). The overlay number of the a-IGZO, ES, and S/D layer is reduced, which resulted in the decrease in the size of TFT device that lowered the parasitic capacitance. The outcome planar structure is similar to the BCE structure (Fig. 3a, b).

Figure 3 shows the SEM images of a-IGZO-based TFTs with CL-ES structure (Fig. 3a, c) and BCE structure (Fig. 3b, d). From the top view, it is difficult to identify the differences between CL-ES structure and BCE structure (Fig. 3a, b). From the side view, an ES nano-layer can be found between the a-IGZO nano-layer and the S/D electrode layer in CL-ES structure (Fig. 3c). Meanwhile, a passivation layer can be found on the top of a-IGZO nano-layer in BCE structure (Fig. 3d). In the presented CL-ES process, an a-IGZO nano-layer with a thickness of 30 nm is deposited. Moreover, the damage during wet etching is negligible. For BCE process, a 70-nm a-IGZO nano-layer is deposited, as a-IGZO layer needs compensation for etching loss. The difference between the thicknesses of a-IGZO nano-layers in CL-ES and BCE structures can be observed in the SEM images (Fig. 3c, d).

**Fig. 4:**
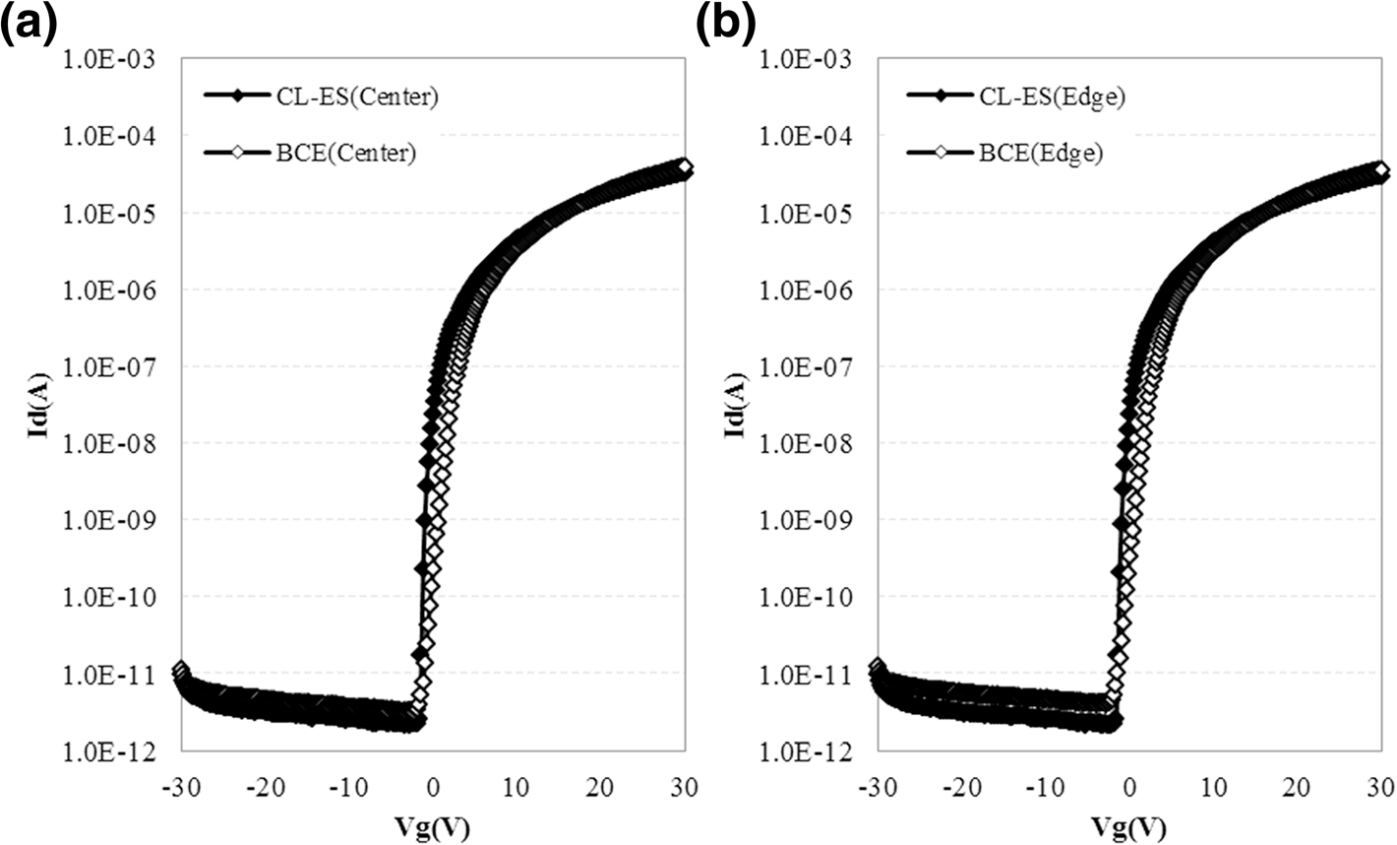
(Color online) Comparison of I-V characteristic of a-IGZO TFTs with CL-ES and BCE structure on the center (**a**) and edge (**b**) of 8.5 generation glass substrate

The I-V characteristics of a-IGZO-based TFT with CL-ES structure and BCE structure are compared (Fig. 4). The saturation electron mobility, threshold voltage, subthreshold voltage swing (SS) value, and more characteristic values are summarized in Table 1. Note that the values summarized in Table 1 are the average number derived from the center and edge of an 8.5 generation glass substrate. The a-IGZO-based TFT with CL-ES structure realizes *V*_th_ of − 0.8 V, SS value of 0.18 V/dec, and saturation electron mobility of 8.05 cm^2^/V s. In the a-IGZO-based TFT with BCE structure, the corresponding results are *V*_th_ of + 0.5 V, SS value of 0.77 V/dec, and saturation electron mobility of 6.03 cm^2^/V s. Compared to the BCE structure, CL-ES structure shows an improved device performances. However, the on-current characteristic of the a-IGZO-based TFT device with CL-ES structure is lower than that with BCE-structured device. This is due to the fact that TFT channel structures are different in CL-ES and BCE structures. Generally, BCE-structured TFT channel length are the distance between S/D metal electrodes, and the measured channel length in this study is 5 um [21]. In CL-ES structure, electrodes are in contact with the a-IGZO nano-film that is stretched at the side of ESL nano-mask. Therefore, the channel length is decided by the distance between the a-IGZOs defined at the etch-stopper’s sides, but not determined by the distance between the electrodes. The channel length of the present CL-ES-structure device is measured to be 10 um.

**Table 1:** Comparison of I-V characteristics of a-IGZO-based TFT device with CL-ES structure and BCE structure

Item	Unit	CL-ES	BCE
*V*_th_Avg_	V	− 0.85	+ 0.50
*V*_th_Range_	V	0.72	2.14
Subthreshold voltage swing	V/dec.	0.18	0.77
*I*_on_/*I*_off_ ratio	–	3.82 × 10^6^	2.62 × 10^6^
Mobility	cm^2^/V s	8.05	6.03

As shown in Table 1, the measured values of *I*_on_/*I*_off_ ratio (~ 10^6^, see Table 1) are approximately 10 times smaller than the typical value (> 10^7^) of a-IGZO-based TFTs. This is because the measuring equipment used here is for the 8.5 generation mass production. Long cables are necessary for these measurements, as the size of the industrial equipment is large. The long cables resulted in an increased measurement noise. In the following reliability testing, smaller-scale measuring equipment is utilized, and the individual TFT devices is used as specimen for measurement. In this way, the measured *I*_on_/*I*_off_ ratios are all upper 10^7^ (see below).

CL-ES process is carefully designed to prevent a-IGZO channel layer being exposed to etchant, photoresist, or stripper. During the process that produces CL-ES process, gate insulator, a-IGZO nano-layer, and ES nano-layer, each inter-layer interface is in contact with only DI water for cleaning purpose. Hence, the chemical contamination is negligible in insulator layer and a-IGZO nano-layer [25, 26]. However, the BCE process not only exposes channel layer to the chemicals but also involves Cu ion diffusion contamination, as the a-IGZO channel is directly exposed to Cu metal. This is also avoided in device with CL-ES structure. The channel region of the a-IGZO nano-film is well protected by ESL nano-mask. The low chemical contamination in CL-ES process may lead to a low carrier trap density at the interface between a-IGZO nano-layer and insulator layer, resulting in an excellent SS value. This low chemical contamination of a-IGZO-based TFT device via CL-ES process also helps improve the uniformity and reproducibility of a-IGZO TFT, which are highly important in industrial production [27, 28].

**Fig. 5:**
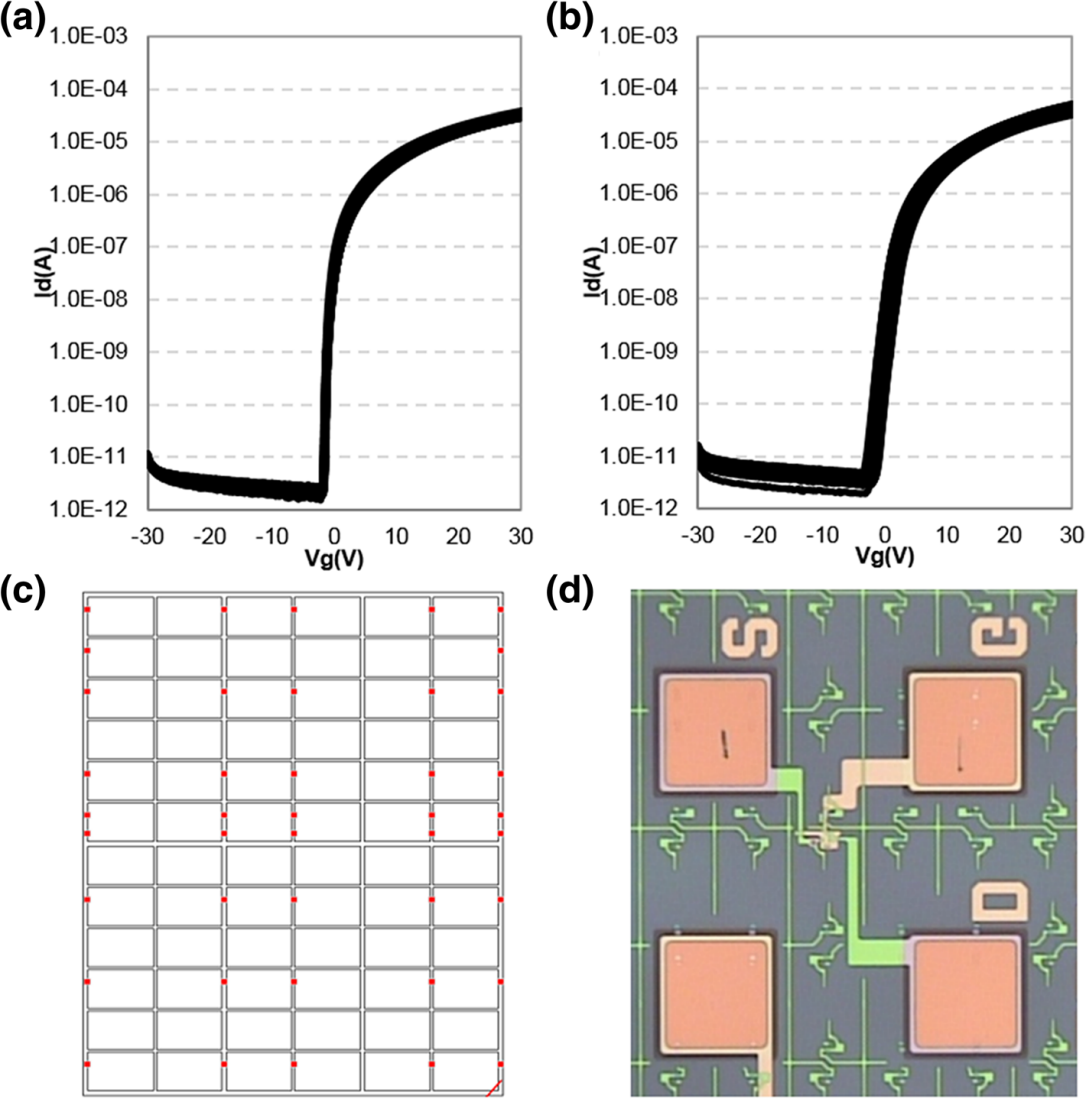
(Color online) **a** CL-ES structure. **b** BCE structure’s TFTs I-V transfer characteristic. **c** 42 measuring points. **d** the photo of TFT. All measured on an 8.5 generation substrate

Figure 5 shows the measured I-V characteristic of TFTs with CL-ES structure and BCE structure derived from 42 measuring points on an 8.5 generation substrate. a-IGZO-based TFT with CL-ES structure has a *V*_th_ range of 0.72 V, while that of BCE-structured device is 2.14 V (Table 1). In other words, the uniformity of device performance is significantly improved by CL-ES structure.

**Fig. 6:**
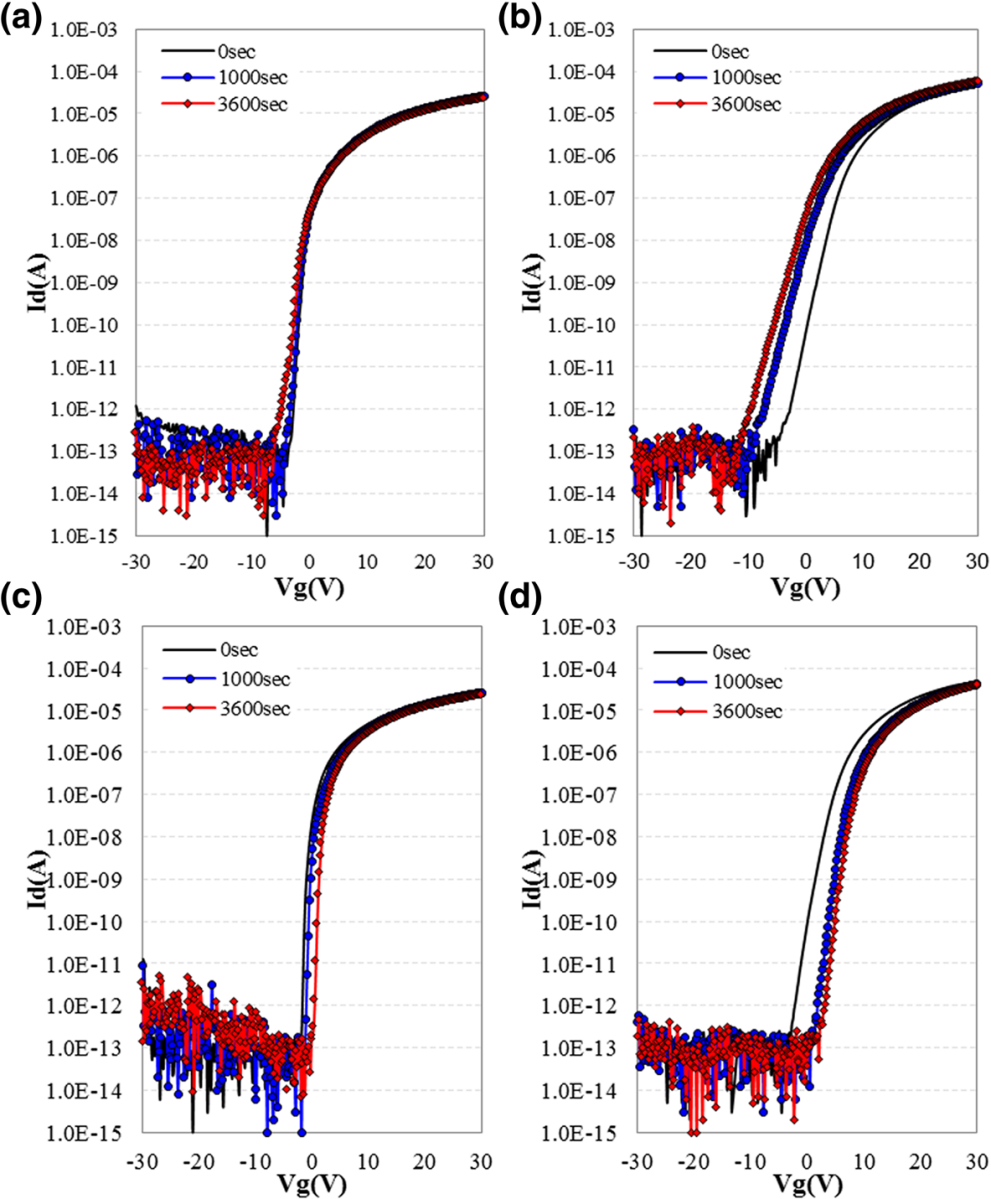
(Color online) I-V transfer characteristic drift of CL-ES (**a**, **c**) and BCE (**b**, **d**) TFT obtained from NBITS (**a**, **b**) and PBTS testing (**c**, **d**)

Figure 6a, b show the I-V characteristic shift of CL-ES-structured device and BCE-structured device obtained in NBTIS testing, respectively. The NBTIS testing results are summarized in Table 2. Under the stress condition described in the Table 2, the *V*_th_ shift of CL-ES-structured device and BCE-structured device are − 0.51 and − 3.88 V, respectively. Additionally, the on-current shift, off-current shift, and SS value variance of the CL-ES-structured device are all lower than those of the BCE-structured device (Table 2); this is because a-IGZO-based device with CL-ES-structure can effectively prevent the contamination of a-IGZO and lower carrier trap density of a-IGZO TFT channel. Especially, when looking at the result from first 1000 s of stress, no SS value change is observed in CL-ES-structured device. This phenomenon is comparable to the 0.16 V/dec increase in SS value of BCE-structured device, as it shows that defect sites, which can form carrier traps on the surface of a-IGZO nano-film constituting CL-ES TFT back channel, are not additionally created by electrical and illumination stress. These results fully prove that CL-ES-structured device is much more stable than BCE-structured device. Figure 6c, d show the I-V curve shift of CL-ES- and BCE-structured TFTs obtained from PBTS testing. The detailed PBTS testing results are summarized in Table 3. Both CL-ES-structured TFT and BCE-structured TFT have decreased in ion current during PBTS evaluation. This is caused by the shift in *V*_th_ to the positive direction. During PBTS evaluation, residual ion current ratio [(last ion/initial ion) × 100] of the CL-ES-structured TFT with relatively smaller *V*_th_ positive shift (+ 1.94 V) is in the level of 88.2%. When compared to the BCE-structured TFT's residual ion current ratio of 41.3%, CL-ES-structured TFT is significantly superior. This shows the important capacity difference during designing of gate drive on array (GOA) circuit. Different from NBTIS, SS value of CL-ES-structured TFT does not have significant variation ((∆SS 0.06 V/dec), or rather decreases (∆SS − 0.86) like as BCE-structured TFT. This is perhaps due to the carriers, accumulate in the inner space and interface between gate insulator and a-IGZO nano-film by positive gate bias, filling the carrier trap site at the early stage, causing decrease in carrier trap phenomenon. Moreover, the threshold voltage shift phenomenon occurs by carrier charge trapped near the interface between gate insulator and a-IGZO nano-film. Small threshold voltage shift of CL-ES-structured TFT represents that the interface and the inner space of a-IGZO are remarkably clean. In conclusion, PBTS testing also suggests that CL-ES structure and process lead to a better device reliability.

**Table 2:** The on-current shift, off-current shift, and subthreshold voltage swing variance values of CL-ES-structured device and BCE-structured device

BL	T	Bias time	CL-ES				BCE			
			*V*_g_ bias − 30 V				*V*_g_ bias − 30 V			
			*I*_on_ (uA)	*I*_off_ (pA)	*V*_th_ (V)	SS (V/dec)	*I*_on_ (uA)	*I*_off_ (pA)	*V*_th_ (V)	SS (V/dec)
0nit	RT	0 s	7.50	0.01	− 0.71	0.50	8.79	0.21	2.34	1.69
5000nit	60 °C	1000 s	7.92	0.12	− 1.06	0.50	11.83	0.57	0.02	1.85
5000nit	60 °C	3600 s	6.99	0.10	− 1.22	0.83	15.99	6.59	− 1.54	2.11
Shift (1 h–0 h)			− 0.51	0.09	− 0.51	0.33	7.20	6.38	− 3.88	0.42

**Table 3:** PBTS testing results of CL-ES-structured device and BCE-structured device

T	Bias time	CL-ES				BCE			
		*V*_g_ bias + 30 V				*V*_g_ bias + 30 V			
		*I*_on_ (uA)	*I*_off_ (pA)	*V*_th_ (V)	SS (V/dec)	*I*_on_ (uA)	*I*_off_ (pA)	*V*_th_ (V)	SS (V/dec)
RT	0 s	8.29	0.10	− 0.32	0.18	10.32	0.14	1.15	1.70
60 °C	1000 s	8.42	0.00	0.14	0.22	5.50	0.20	5.73	0.93
60 °C	3600 s	7.32	0.19	1.62	0.24	4.26	0.07	6.73	0.84
Shift (1 h–0 h)		− 0.97	0.09	1.94	0.06	− 6.06	− 0.06	5.58	− 0.86

## Conclusions

In conclusion, a newly developed CL-ES process has been successfully developed to fabricate a-IGZO-based TFT backplane with five masks for advanced display. The CL-ES process has the advantages of an etch-stopper-layer structure while maintaining the equal number of masks and similar device areas to a BCE process, which overcomes the problem of increased mask number and occupied area in conventional etch-stopper TFT devices. A newly formed ESL nano-mask and a simultaneous etching of a-IGZO nano-layer and S/D electrode nano-layer enable a high uniformity and stability of device for large-area display. In respect of electrical performance, the reproducibility and reliability of device performance of a-IGZO-based TFT with CL-ES structure are much better than that of BCE-structured device. The a-IGZO-based TFT device has a *V*_th_ distribution over 42 measuring points TFTs on the 8.5 generation glass substrate of 0.72 V, saturation electron mobility of 8.05 cm^2^/V s, and SS value of 0.18 V/dec. According to reliability evaluation results obtained from NBTIS and PBTS, *V*_th_ variances before and after stress of CL-ES a-IGZO-based TFTs are − 0.51 and 1.94 V after 3600 s of stress, respectively. The SS value variances are 0.33 and 0.06 V/dec. Therefore, by overcoming the technological and economic obstacles, the presented CL-ES technique will pave the way for next-generation high-resolution and large panel display products.

## Abbreviations

a-IGZO: Amorphous indium-gallium-zinc-oxide
AM-LCD: Active matrix liquid crystal display
BCE: Back channel etch
ESL: Etch-stopper layer
GOA: Gate drive on array
NBTIS: Negative bias temperature illumination stress
PBTS: Positive bias temperature stress
SiNx: Silicon nitride
SiOx: Silicon oxide
SS: Subthreshold swing
TFT: Thin film transistor
TN LCD: Twisted nematic liquid crystal display
